# Effect of reduced crude protein diets supplemented with free limiting amino acids on body weight, carcass yield, and breast meat quality in broiler chickens

**DOI:** 10.1016/j.psj.2023.103041

**Published:** 2023-08-18

**Authors:** Sahar Benahmed, Amani Askri, Théophane de Rauglaudre, Marie-Pierre Létourneau-Montminy, Nabeel Alnahhas

**Affiliations:** Department of Animal Science, Faculty of Agricultural and Food Sciences, Université Laval, Quebec City G1V 0A6, Quebec, Canada

**Keywords:** broiler chicken, meat quality, protein functionality, reduced crude protein

## Abstract

This study investigated the effect of reducing dietary crude protein (**CP**) content in the grower and finisher diets of broiler chickens on breast meat quality, muscle protein functionality, growth, carcass yield, and meat yield. To achieve this, a total of 1,269 one-day-old male Ross 308 chicks were fed 1 of 3 diets replicated 9 times each in a randomized complete block design with 9 blocks. The diets included a control (20.4% and 19.5% CP in the grower and finisher phase, respectively), a diet with a 1.5% reduction (CP-1.5%) and a diet with a 3.0% reduction (CP-3.0%) in CP content in both the grower and finisher phases. At the end of the experiment, the reduced-CP diets had no impact on body weight, feed intake, or feed conversion ratio. However, reduced-CP diet resulted in reduced (*P* < 0.001) total nitrogen intake (−7.46 and −11.94% in CP-1.5% and CP-3.0%, respectively). Breast meat quality was assessed (n = 36 birds/group), and the experimental diets were associated with a slightly increased (*P* = 0.07) ultimate pH (5.75, 5.79, and 5.81 for the control, CP-1.5%, and CP-3.0%, respectively). Breast fillets from the CP-1.5% and CP-3.0% groups had lower yellowness (b*, *P* < 0.001) and lower cooking loss (CL, *P* < 0.001) values than the control. Moreover, the solubility, emulsion activity, and stability indices of the sarcoplasmic and myofibrillar fractions of muscle proteins were not influenced by the diets. CP-1.5% and CP-3.0% diets were associated with an increased (*P* < 0.001) breast yield (18.39, 19.21, and 19.61% for the control, CP-1.5%, and CP-3.0%, respectively) while leg yield remained unchanged. Additionally, breast meat nutritional properties including protein and lipid contents were not impacted by the experimental diets. In conclusion, the CP content in the grower and finisher diets of broiler chickens can be reduced by as much as 3.0% without detrimental effects on performance or on meat quality as long as birds' amino acid requirements are adequately met.

## INTRODUCTION

Global demand of poultry meat is steadily increasing. In 2020, poultry accounted for 41% of global meat consumption, and is expected to account for 52% of global meat consumption by 2030 (OECD, 2022). Strategies are required to improve the sustainability of poultry meat production to meet the increasing global demand with limited impact on the environment. Reducing crude protein (**CP**) content in broilers’ diets represents one such strategy that reduces nitrogen (**N**) losses and ammonia (**NH_3_**) emissions (Chalova et al., 2016; Liu et al., 2021). Reducing CP from 22.4 to 17.9% in mixed-sex broilers’ diets induced a 19.2% reduction in nitrogen excretion from 1.2 g/bird/d to 0.97 g/bird/d (Lemme et al., 2019). A recent meta-analysis of the available literature has also revealed that reducing CP content from 19% to 17% in the diets of broilers aged 0 to 21 d was associated with 29% reduction in nitrogen excretion and a 7% increase in nitrogen retention relative to nitrogen intake (Alfonso-Avila et al., 2022). Reducing dietary CP content also has beneficial effects on broiler intestinal health because of the reduction of undigested protein flow to the large intestine, which limits the proliferation of opportunistic pathogens (Greenhalgh et al., 2020). Animal welfare is also improved, and a linear relationship has been evidenced between reduced dietary CP content (from 20.8 to 17.8%) and decreased litter moisture, significantly decreasing the average score of footpad dermatitis from 143 at 20.8% CP to 39 at 17.8% CP (van Harn et al., 2019). The reduce litter humidity is probably a result of reduced water intake in birds receiving reduced-CP diets. It has been estimated that a reduction of CP content from 19% to 17% decreased daily water intake by 20.6 mL/bird and reduced litter humidity by 2.2% (Alfonso-Avila et al., 2022).

The effects of reduced-CP diets on growth performance, feed efficiency, and breast meat yield have also been investigated. For instance, reducing CP in diets of male broilers from 19% to 17% did not impair body weight gain, final body weight at 35 d, feed intake, or feed conversion ratio (Belloir et al., 2017). These authors also found that although reducing CP in the above-mentioned range did not impact breast meat yield, it led to a slight but significant increase in the abdominal fat percentage from 2.16% at 19% CP to 2.45% at 17% CP. Similar findings were also reported by Lambert et al. (2022) who used experimental diets with CP content ranging from 18.9 to 17.1% in the grower phase and from 17.1% to 15.3% in the finisher phase and reported no significant impact on growth performance or meat yield. It is important to note that in these studies, the requirements in amino acids (**AA**) were adequately met by supplementing reduced-CP diets with synthetic AA. In a recent meta-analysis, de Rauglaudre et al. (2023) showed that reducing CP by an average of 1.62% and up to a maximum of 3.22% while AA requirements were adequately met was associated with maintained growth performance and nitrogen retention.

These data suggest that CP content can be reduced by up to 3.0%, leading to a significant reduction in nitrogen and ammonia emissions without compromising growth performance and associated traits of economic importance for the industry as long as requirements in AA are met. However, before recommending reduced-CP diets for use in practice, their impact on meat quality needs to be investigated. As mentioned before, reduced-CP diets are often supplemented with unbound AA to provide birds with their AA requirements (Liu et al., 2021), and variation in the levels of these AA could alter the final meat quality by altering muscle glycogen stores and its postmortem metabolism (Berri et al., 2008; Guardia et al., 2014; Belloir et al., 2019). Other than the effect of variations in dietary AA content on meat quality traits, little is known about the effect of reduced dietary CP contents on the technological traits of broiler breast meat. The aim of this study was thus to investigate the effect of reduced-CP diets in the grower and finisher phases on 1) the technological quality traits of breast meat, 2) some functional properties of muscle proteins, and 3) growth performance and carcass yield.

## MATERIALS AND METHODS

Experimental and animal care procedures were reviewed and approved by the Institutional Animal Care and Use Committee of Université Laval according to the guidelines of the Canadian Council on Animal Care (Project #2022-1122).

### Birds, Experimental Design, and Housing

A total of 1,269 one-day-old male Ross 308 chicks were obtained from a local commercial hatchery (Scott, Quebec, Canada) and placed in an experimental poultry house (Animal Science Research Center, Deschambault, Quebec, Canada). Chicks were distributed in a randomized complete block design with 3 treatments in 9 blocks. Each treatment was replicated 9 times with 47 chicks placed in each floor pen (3.6 m^2^). The pens were equipped with bell drinkers and a manual feeder, and the floor was bedded with sawdust (24 kg/pen). Ambient temperature was maintained at 33°C during the first week, gradually reduced to 22°C by the end of the third week, and then maintained at 22°C to the end of the experiment at 35 d. A lighting program meeting the requirements of Ross 308 chicks was applied during this experiment.

### Experimental Diets

During the starter phase (0–10 d), all birds received the same corn soybean meal–based diet (21.39% CP and 2,950.1 Kcal/kg of ME). The experimental diets were distributed during the grower (11–22 d) and the finisher (23–35 d) phases (Table 1). In both experimental phases, the diets included a control group (20.4% and 19.5% of CP for the grower and finisher phase, respectively), a group with a 1.5% reduction in CP content (CP-1.5%), and a group with a 3.0% reduction in CP content (CP-3.0%). As can be seen in Table 1, the dietary CP content was reduced by partially replacing soybean meal with corn and canola meal in both the grower and finisher diets. To ensure that the observed effects were only due to differences in CP levels, all diets in each phase were formulated to have the same digestible lysine content. Synthetic AA were also added to the formulated diets to meet birds’ AA requirements. All diets were formulated to meet birds’ nutritional requirements according to the Brazilian tables for poultry and swine nutritional requirements (Rostagno et al., 2017).

**Table 1:** Ingredient composition of the experimental grower (11–22 d) and finisher (23–35 d) diets.

Ingredients, %	Grower diets^1^			Finisher diets		
	Control	CP-1.5%	CP-3.0%	Control	CP-1.5%	CP-3.0%
Corn	52.45	55.50	59.05	55.07	58.78	62.37
Wheat	10.00	10.00	10.00	10.00	10.00	10.00
Corn DDGS	5.00	5.00	5.00	5.00	5.00	5.00
Corn gluten, 60%	4.00	2.50	0.00	4.00	2.50	0.00
Soybean meal	23.00	19.00	16.70	21.10	18.30	15.00
Canola meal	2.20	4.20	4.70	1.70	2.00	3.50
Soybean oil	0.00	0.00	0.00	0.10	0.00	0.00
Lime stone	1.18	1.18	1.20	1.06	1.08	1.10
Monocalcium phosphate	0.86	0.86	0.86	0.68	0.70	0.70
Salt	0.30	0.30	0.30	0.30	0.30	0.29
Sodium bicarbonate	0.14	0.24	0.32	0.14	0.14	0.16
Vit E, 50 000 IU	0.07	0.07	0.07	0.06	0.06	0.06
Vitamin premix	0.02	0.02	0.02	0.02	0.02	0.02
Liquid choline, 75%	0.05	0.05	0.05	0.05	0.05	0.05
Trace minerals premix	0.10	0.10	0.10	0.09	0.09	0.09
Lysine sulfate, 70%	0.30	0.44	0.56	0.32	0.44	0.56
DL-Methionine, 99%	0.17	0.20	0.26	0.15	0.20	0.25
Threonine, 98%	0.08	0.14	0.20	0.08	0.13	0.20
Tryptophane, 98%	0.00	0.00	0.00	0.00	0.00	0.00
L-Isoleucine, 98.5%	0.00	0.02	0.09	0.00	0.02	0.10
L-Arginine, 98.5%	0.00	0.06	0.15	0.00	0.07	0.17
L-Glycine, 98%	0.00	0.00	0.15	0.00	0.00	0.16
L-Histidine-HCL, 98.5%	0.00	0.00	0.02	0.00	0.00	0.02
L-Valine, 96.5%	0.00	0.03	0.12	0.00	0.03	0.12
Xylanase	0.05	0.05	0.05	0.05	0.05	0.05
Phytase	0.04	0.04	0.04	0.04	0.04	0.04

1 Control: 20.4% CP in grower and 19.5% CP in finisher diets, CP-1.5%: – 1.5% CP, CP-3.0%: – 3.0% CP.

### Body Weight and Feed Intake

Per pen body weight was recorded at 0, 11, 21, and 35 d while feed intake was measured at 11, 21, and 35 d to calculate the feed conversion ratio (**FCR**) and total nitrogen intake (**NI**). NI was calculated (g/bird) according to Belloir et al. (2017) using the following formula:

$$NI\;\left(g/bird\right)=\frac{\left(FI\;\left(g\right)\;\times \;NcS\;\left(\frac{g}{kg}\right)\right)\;+\left(FI\;\left(g\right)\;\times \;NcG\;\left(\frac{g}{kg}\right)\right)\;+\;\left(FI\;\left(g\right)\;\times \;NcF\;\left(\frac{g}{kg}\right)\right)}{1000}$$

where FI is the feed intake (g/bird); and NcS, NcG, and NcF are the dietary nitrogen content (g/kg) in the starter, the grower, and the finisher diets, respectively. The dietary N content was calculated as N (g/kg) = (CP% in diet ÷ 6.25) × 10. The per pen FCR was calculated for the whole experiment (0 d to 35 d) using the following formula:

$$FCR=\frac{FI\;\left(\frac{kg}{pen}\right)}{WG\;\left(\frac{kg}{pen}\right)}$$

where FI is the total feed intake and WG is the weight gain between 0 d and 35 d.

### Processing and Meat Quality Measurements

At 30 d, a total of 108 birds (4/pen/treatment) were randomly selected and individually identified using leg bands. At 35 d and after 8 h of feed withdrawal, identified birds were individually weighted, placed in cages, and transported (< 2 h) to a small-scale commercial processing unit (Adstock, Quebec, Canada) where they were processed according to commercial practices. After processing, carcasses were cut and individually placed in plastic bags and brought back in coolers on ice to the Food Science laboratory (Faculty of Agricultural and Food Sciences, Université Laval, Quebec, Canada) where they were immediately transferred to a cold room (4 °C). Next, carcass weight and weight of individual cuts (whole breast, the Pectoralis major, the Pectoralis minor, whole thigh, upper thigh, drumstick, and wings) were recorded. The yield of the whole carcass and that of carcass cuts were evaluated relative to body weight (**BW**) at 35 d and expressed as percentages.

After 24 h at 4 °C, the ultimate pH (**pHu**) and the color parameters including lightness (L*), redness (a*), and yellowness (b*) were measured on the dorsal (bone side) surface of the cranial (the thickest) part of the Pectoralis major muscle as described previously (Alnahhas et al., 2014). The cooking loss (**CL**) was measured by taking a muscle sample (average weight of 60 ± 2 g) from the middle part of breast fillets, placing in a plastic bag (Whirl-Pak bag, Nasco Whirl-Pak, VWR, Otario, Canada), and cooking it in a water bath at 85 °C until an internal temperature of 76 °C was reached. After cooking, samples were cooled in an ice bath for 10 min, taken out of their bags, wiped gently with an absorbent paper, and weighed again. The cooking loss was expressed as a percentage of the sample's initial weight before cooking. Cooked meat samples were then cut into strips (1 cm × 1 cm × 3 cm) parallel to the direction of muscle fibers to evaluate their shear force using a texture analyzer (ZwickiLine, Zwick/Roell, Germany) equipped with a Warner-Bratzler blade moving at a crosshead speed of 220 mm/min. This test was performed in triplicate, and the average of the maximum force (N/cm^2^) required to shear the replicates was reported.

### Proximate Composition

Samples (n = 13/treatment) were also taken from the cranial and caudal part of the muscle to measure their moisture, protein, and lipid contents according to Official Methods (AOAC, 2023). First, samples were lyophilized (model 50L Virtual EL-85, VirTis, Los Angeles, CA). Then, the cranial and caudal parts of each sample were homogenized using a grinder (Knife Mill Grindomix GM 300, Haan NRW, DE) at a speed of 2,000 rpm for 30 s. A unique sample per muscle was taken for all analyses.

### Functional Properties of Muscle Proteins

Solubility of muscle proteins was measured on samples (n = 13/treatment) taken from the cranial part of the Pectoralis major at 24 h postmortem using a previously described method (Bowker and Zhuang, 2016). Briefly, duplicate 1 g samples were homogenized in 20 mL ice-cold 25 mM potassium phosphate buffer (pH 7.2). The homogenates were centrifugated at 15,300 *× g* for 15 min at 4°C, and the supernatant was carefully transferred to a new tube through a Whatman N°1 filter paper. The solubility of the sarcoplasmic fraction was determined by measuring the protein concentration of this supernatant using a BCA assay (Pierce™ BCA Protein Assay Kit, Thermo Fisher Scientific) with a BSA standard curve. The solubility of the myofibrillar fraction was similarly determined after homogenizing the remaining pellet in 20 mL ice-cold buffer (0.55 M KI, 50 mM potassium phosphate at pH 7.2), centrifuging at 15,300 *× g* for 15 min at 4°C and pouring the supernatant carefully into a new tube through a Whatman N°1 filter paper.

Next, the emulsion activity index (**EAI**) was determined on the same samples according to a previously described method (Chan et al., 2011). Briefly, after determining the protein concentration of the sarcoplasmic and myofibrillar fractions as described above, it was adjusted to 1.5 mg/mL in both fractions, mixed with corn oil in a 3:1 ratio (volume/volume), and homogenized at 12,000 rpm for 1 min using an Ultraturrax (IKA, Wilmington, NC). Aliquots of the emulsions (35 µL) were diluted to 3.5 mL in 0.1% SDS buffer in quadruplicate. Immediately after dilution, the turbidity was read at 500 nm using a spectrophotometer (Genesys 10S UV-Vis, Thermo Scientific). The EAI was then calculated as EAI = 2.33 × A_0_, where A_0_ is the absorbance of the emulsion. To determine the emulsion stability index (**ESI**), the absorbance of the emulsions was read a second time after settling at room temperature for 20 min without agitation. The ESI was then calculated as ESI = 10 × [A_0_/(A_0_−A_20_)], where A_0_ is defined as before and A_20_ is the absorbance measured 20 min after A_0_.

Finally, the myofiber fragmentation index (**FIdx**) was measured (n = 13 samples/treatment) according to the method described previously in (Thompson et al., 1987). Briefly, frozen 1 g muscle samples were homogenized on ice in 10 mL of ice-cold buffer (0.25 M sucrose and 0.02 M KCl) for 30 s at high-speed using an Ultraturrax. The homogenates were vacuum aspirated through a tared 250 µm nylon screen (Tong Gu, China) to visible dryness. After air drying for 10 min on an absorbent paper, the nylon screen with the residue was weighed again, and the FIdx was calculated as FIdx = weight of nylon screen with the residue/original sample weight × 1000. According to this method, a higher FIdx value is indicative of lower fragmentation of myofibers.

### The Allometric Growth Coefficient of Carcass Cuts

The allometric growth coefficients of carcass cuts were determined using the following equation:

$$l_{Y}=\;a+b.l_{X}$$

where $l_{Y}$ is the natural logarithm of the weight of carcass cuts, $l_{X}$ is the natural logarithm of carcass weight, a is the constant (i.e., intercept) of the equation, and b is the allometric coefficient (i.e., the slope) of the relationship between the weight of the carcass cuts and that of the carcass (Sousa et al., 2019). If b = 1, then the carcass part (Y) grows at the same rate as the carcass (X) and growth is said to be isometric. If b > 1 or b < 1, Y is growing faster or slower than X, respectively (Zuidhof et al., 2014).

### Statistical Analysis

The effect of treatments on measured traits was analyzed using a linear mixed effects model fitted to the data using the R package *lmerTest* (Kuznetsova et al., 2017). The model included the treatment as a fixed effect while the effect of the block and the pen intra-block were fitted as random effects. The results were reported as the least squares means and their standard errors. Linear and quadratic contrasts and differences between treatment means were tested for significance, and *P*-values were adjusted according to the Tukey method as implemented in the *emmeans* package of R (R Core Team, 2020). Treatment effects and contrasts were considered statistically significant at *P* < 0.05.

To test if the allometric coefficients were (b = 1), (b > 1), or (b < 1), and to compare the slopes of different treatments, their 95% confidence intervals (95% CI) were computed from the estimated standard errors of these coefficients, as obtained from the regression model described above. An allometric coefficient was considered significantly different from unity at *P* < 0.05 if its CI did not overlap with unity, and 2 allometric coefficients were considered significantly different at *P* < 0.05 when their respective CI did not overlap. The data were log-transformed using the *log* function, and the models were fitted to the log-transformed data using the *lm* function in R version 4.0.2 (R Core Team, 2020).

## RESULTS

In the grower diets, the calculated and analyzed levels of CP were generally in good accordance (Table 2). The CP-1.5% and CP-3.0% treatment had slightly greater (+0.8 percentage points) analytical CP content than the calculated values (Table 2). A slight discordance between the calculated and analyzed values was found in the finisher diets, with the analyzed CP levels being 0.80, 1.1, and 1.2 percentage points higher than the calculated values in the control, CP-1.5%, and CP-3.0% group, respectively. Based on the analyzed values of CP in the grower diets, the CP content was lower than the control group by 0.7 and 2.2 percentage points in the CP-1.5%, and CP-3.0% group, respectively. In the finisher diets, the achieved CP reduction compared with the control group was 1.2 and 2.6 percentage points in the CP-1.5% and CP-3.0% group, respectively.

**Table 2:** Formulated nutrient composition and analytical crude protein content of the experimental grower (11–22 d) and finisher (23–35 d) diets.

Nutrients	Grower diets^1^			Finisher diets		
	Control	CP-1.5%	CP-3.0%	Control	CP-1.5%	CP-3.0%
Calculated CP, %	20.4	19.0	17.5	19.5	18.0	16.5
Analytical CP, %	20.5	19.8	18.3	20.3	19.1	17.7
ME, kcal/kg	3,010	3,010	3,010	3,050	3,057	3,055
Phytase, FTU/kg	600	600	600	600	600	600
Available phosphate, %	0.44	0.44	0.43	0.40	0.40	0.40
Calcium, %	0.72	0.72	0.72	0.63	0.63	0.64
Chloride, %	0.24	0.24	0.24	0.24	0.24	0.24
Phosphate, %	0.56	0.55	0.54	0.51	0.50	0.49
Potassium, %	0.79	0.73	0.70	0.75	0.70	0.66
Sodium, %	0.18	0.21	0.23	0.18	0.18	0.18
DEB^2^, mEq/kg	212	210	210	202	190	181
dLys, %	1.00	1.00	1.00	0.96	0.95	0.95
Met+Cys/dLys	0.75	0.75	0.75	0.75	0.75	0.75
Methionine/dLys	0.48	0.49	0.51	0.47	0.49	0.51
Threonine/dLys	0.70	0.70	0.70	0.70	0.70	0.70
Tryptophan/dLys	0.21	0.19	0.18	0.21	0.19	0.17
Arginine/dLys	1.09	1.05	1.05	1.07	1.05	1.06
Histidine/dLys	0.43	0.39	0.37	0.43	0.40	0.37
Isoleucine/dLys	0.74	0.69	0.67	0.73	0.68	0.67
Leucine/dLys	1.71	1.52	1.26	1.73	1.55	1.28
Valine/dLys	0.84	0.80	0.80	0.84	0.80	0.80
Phe+Tyr/dLys	1.53	1.36	1.14	1.53	1.36	1.13
Glycine + serine/dLys	1.50	1.37	1.35	1.49	1.35	1.36

1 Control: 20.4% CP in grower and 19.5% CP in finisher diets, CP-1.5%: – 1.5% CP, CP-3.0%: – 3.0% CP.

2 Dietary electrolytic balance.

### Effect of Reducing Crude Protein on the Performances

Over the whole period of the experiment (0–35 d), the impact of reducing dietary CP content in the grower and finisher diets by 1.5% or 3.0% was not statistically significant for BW, weight gain, feed intake, and FCR (Table 3). On the contrary, birds that received either of the 2 experimental diets consumed a similar amount of nitrogen that was significantly lower than that consumed by birds from the control group.

**Table 3:** Effect of dietary treatments on body weight gain, feed intake, feed conversion ratio, and total nitrogen intake^1^ from 0 to 35 d.

Trait	Treatment^2^			*P*-value^3^			
	Control	C-1.5%	C-3.0%	TRT	L	Q	C vs. CP
BW, g	2,575.0 ± 31.7	2,583.0 ± 31.7	2,579.0 ± 31.7	0.98	0.93	0.87	0.46
Weight gain, g/bird	2,530.0 ± 31.5	2,538.0 ± 31.5	2,534.0 ± 31.5	0.97	0.91	0.85	0.85
FI, g/bird	4,103.0 ± 86.0	3,993.0 ± 86.0	4,054.0 ± 86.0	0.66	0.69	0.42	0.45
FCR	1.59 ± 0.03	1.54 ± 0.03	1.57 ± 0.03	0.47	0.54	0.29	0.29
NI, g/bird	134.0 ± 2.70^a^	124.0 ± 2.70^b^	118.0 ± 2.70^b^	< 0.001	< 0.001	0.65	< 0.001

1 Values are least squares means of 9 pens per treatment ± the standard error. FI: feed intake, FCR: Feed conversion ratio, NI: total nitrogen intake.

2 Control: control diet (20.4% CP and 19.5% CP in the grower and finisher diets, respectively), CP-1.5%: −1.5% CP, CP-3.0%: −3.0% CP.

3 TRT is the *P*-value of the effect of treatment on analyzed traits; L and Q are the *P*-values of the linear and quadratic contrasts, respectively. C vs*.* CP is the *P*-value of the contrast comparing the average of the 2 experimental diets against the control diet. Means within a row lacking a common superscript differ at *P* < 0.05.

### Effect of Reducing Crude Protein on Body Weight at Slaughter and on Carcass Yield

Final BW (**BWf**) at slaughter and carcass yield were not affected by CP reduction (Table 4). Reduced dietary CP content resulted in a significant increase in breast meat yield (**BMY**) relative to BWf when compared to the control diet. Further analysis of the yield of the 2 pectoral muscles separately revealed that the increase in BMY resulted from a significant increase in the yield of the Pectoralis major muscle, while the yield of the Pectoralis minor muscle did not differ between groups. For BMY and for the yield of the Pectoralis major, the linear contrast was significant while the quadratic was not significant. In addition, the specific contrast comparing the control group with the average of the 2 experimental diets (C vs. CP) was also statistically significant. When evaluated relative to empty carcass weight, the BMY followed the same trend as when it was evaluated relative to BWf. The yield of the leg, upper thigh, and drumstick did not differ between treatment groups. Finally, the yield of the wings in the CP-3.0% group was slightly but significantly reduced (−0.45 percentage points) compared with the control group, and the linear contrast as well as the C vs. CP contrast were also statistically significant for this trait.

**Table 4:** Effect of dietary treatments on body weight at slaughter, carcass yield, and the yield of carcass cuts.^1^

Trait^2^	Treatments^3^			*P*-value^6^			
	Control	CP-1.5%	CP-3.0%	TRT	L	Q	C vs. CP
BWf, g	2,498.09 ± 45.87	2,532.04 ± 45.87	2,534.74 ± 45.87	0.79	0.55	0.75	0.50
Carcass^4^, %	72.89 ± 0.38	72.64 ± 0.38	73.09 ± 0.40	0.74	0.73	0.49	0.95
BMY†, %	18.39 ± 0.31^b^	19.21 ± 0.32^a^	19.61 ± 0.33^a^	< 0.001	< 0.001	0.56	< 0.001
P. major†, %	15.62 ± 0.28^b^	16.38 ± 0.28^a^	16.71 ± 0.29^a^	< 0.001	< 0.001	0.49	< 0.001
P. minor†, %	2.77 ± 0.07	2.82 ± 0.07	2.89 ± 0.07	0.37	0.18	0.84	0.27
BMY§, %	25.19 ± 0.33^b^	26.39 ± 0.33^a^	26.78 ± 0.33^a^	< 0.01	< 0.01	0.34	< 0.01
Leg^5^, %	28.02 ± 0.24	27.94 ± 0.24	27.85 ± 0.26	0.89	0.65	0.99	0.69
Upper thigh, %	18.05 ± 0.21	17.94 ± 0.21	18.09 ± 0.22	0.87	0.89	0.62	0.89
Drumstick, %	9.97 ± 0.11	10.00 ± 0.11	9.76 ± 0.12	0.29	0.22	0.35	0.52
Wings, %	8.11 ± 0.11^a^	7.94 ± 0.11^ab^	7.66 ± 0.12^b^	< 0.05	< 0.01	0.59	< 0.05

1 Values are least squares means of 36 samples per treatment ± the standard error.

2 BWf: body weight of processed birds, BMY: breast meat yield, P. major and P. minor: yield of the pectoralis major and minor, respectively.

† Yield relative to BWf.

§ yield relative to carcass weight.

3 Control: control diet (20.4% CP and 19.5% CP in the grower and finisher diets, respectively), CP-1.5%: −1.5% CP, CP-3.0%: −3.0% CP.

4 Eviscerated carcass yield.

5 Leg included the drumstick, the upper thigh, and half of the corresponding vertebral column.

6 TRT is the *P*-value of the effect of treatment on analyzed traits; L and Q are the *P*-values of the linear and quadratic contrasts, respectively. C vs. CP is the *P*-value of the contrast comparing the average of the 2 experimental diets against the control diet. Means within a row lacking a common superscript differ at *P* < 0.05.

### Effect of Reducing Crude Protein on Yield Dynamics of Carcass Cuts

In the present study, the allometric coefficients (b) of carcass cuts did not differ between the experimental groups. Moreover, only the allometric coefficient for the wings in the CP-1.5% group was significantly different (lower) from unity (Table 5).

**Table 5:** Allometric coefficients^1^ of carcass cuts relative to carcass weight.

Carcass cut^2^	Control			CP-1.5%			CP-3.0%		
	b	SE	R^2^	b	SE	R^2^	b	SE	R^2^
Breast	1.02	0.15	0.69	0.90	0.13	0.56	1.16	0.13	0.73
P. major	1.07	0.15	0.68	0.92	0.13	0.58	1.24	0.14	0.71
P. minor	0.77	0.27	0.22	0.80	0.24	0.18	0.69	0.24	0.25
Leg	1.02	0.09	0.77	1.00	0.08	0.88	1.00	0.08	0.85
Upper thigh	1.17	0.12	0.72	1.02	0.11	0.76	1.07	0.11	0.80
Drumstick	0.76	0.13	0.50	0.95	0.11	0.71	0.87	0.11	0.73
Wings	0.74	0.14	0.50	**0.70**	0.12	0.52	0.79	0.12	0.52

1 Allometric coefficients (b), their standard error (SE), and the proportion of variance explained by the regression model fitted to the log-transformed data (R^2^).

2 Based on their 95% confidence interval, allometric coefficients in italics are significantly higher than unity while those in bold are significantly lower than unity.

### Effect of Reducing Crude Protein on Breast Meat Quality, the Functional Properties of Breast Muscle Proteins, and the Proximate Composition of Breast Meat

The current results did not reveal any significant differences between treatment groups in breast meat quality traits except for b* and CL (Table 6). The yellowness (b*) of breast fillets decreased significantly with reduced dietary CP content. The linear contrast for this trait was statistically significant, but the quadratic contrast was not. In addition, the average values of b* in the 2 groups that received the experimental diets were significantly lower than b* of the control group. Cooking loss also decreased linearly and significantly in breast fillets of broilers fed the reduced-CP diets compared with the control. It is worth noting that the changes in PM-pHu and the Warner-Bratzler shear force (**WBSF**) tended to be significant, with PM-pHu tending to increase and WBSF tending to decrease with reduced CP content. The linear and the C vs. CP contrasts for both traits were statistically significant.

**Table 6:** Effect of dietary treatments on breast meat quality traits.^1^

Trait^2^	Treatments^3^			*P*-value^4^			
	Control	CP-1.5%	CP-3.0%	TRT	L	Q	C vs. CP
PM-pHu	5.75 ± 0.02	5.79 ± 0.02	5.81 ± 0.02	0.07	< 0.05	0.51	0.03
L*	57.56 ± 0.55	57.29 ± 0.55	57.74 ± 0.57	0.76	0.77	0.51	0.94
a*	6.86 ± 0.48	7.2 ± 0.49	6.59 ± 0.50	0.57	0.64	0.34	0.94
b*	15.67 ± 0.39^a^	13.67 ± 0.39^b^	12.1 ± 0.41^c^	< 0.001	< 0.001	0.66	< 0.001
CL, %	17.03 ± 0.28^a^	16.22 ± 0.28^ab^	15.28 ± 0.30^c^	< 0.001	< 0.001	0.86	< 0.01
WBSF, N/cm^2^	27.60 ± 1.12	24.87 ± 1.13	24.60 ± 1.16	0.06	< 0.05	0.28	< 0.05

1 Values are least squares means of 36 samples per treatment ± the standard error.

2 PM-pHu: Ultimate pH of the Pectoralis major muscles, L*: lightness, a*: redness, b*: yellowness, CL: Cooking loss, WBSF: Warner-Bratzler shear force.

3 Control: control diet (20.4% CP and 19.5% CP in the grower and finisher diets, respectively), CP-1.5%: −1.5% CP, CP-3.0%: −3.0% CP.

4 TRT is the *P*-value of the effect of treatment on analyzed traits, L and Q are the *P*-values of the linear and quadratic contrasts, respectively. C vs. CP is the *P*-value of the contrast comparing the average of the 2 experimental diets against the control diet. Means within a row lacking a common superscript differ at *P* < 0.05.

The solubility of the myofibrillar and sarcoplasmic proteins of the Pectoralis major muscle at 24 h postmortem was not influenced by the experimental diets (Table 7). The emulsion activity and stability indices of both protein fractions did not differ between treatment groups. Reducing dietary CP content in the grower and finisher diets had no significant impact on the fragmentation index of muscle fibers. Finally, reducing dietary CP content in the grower and finisher diets was not associated with significant changes in the proximate composition of the breast meat (Table 8).

**Table 7:** Effect of dietary treatments on the functional properties of muscle proteins.^1^

Trait^2^	Treatments^3^			*P*-value^4^			
	Control	CP-1.5%	CP-3.0%	TRT	L	Q	C vs. CP
FIdx	326.36 ± 34.73	258.18 ± 38.83	260.68 ± 38.83	0.33	0.28	0.53	0.21
	Myofibrillar proteins						
Solubility, mg/g	125.32 ± 6.34	133.2 ± 7.64	124.08 ± 7.29	0.52	0.78	0.38	0.78
EAI	2.81 ± 0.06	2.78 ± 0.07	2.85 ± 0.07	0.89	0.76	0.76	0.93
ESI	35.83 ± 2.94	39.08 ± 3.54	37.99 ± 3.37	0.79	0.65	0.75	0.57
	Sarcoplasmic proteins						
Solubility, mg/g	69.8 ± 4.37	76.87 ± 4.98	72.99 ± 4.60	0.64	0.67	0.44	0.43
EAI	0.96 ± 0.04	0.89 ± 0.05	0.93 ± 0.05	0.59	0.77	0.44	0.50
ESI	11.88 ± 0.15	11.66 ± 0.17	11.7 ± 0.16	0.54	0.35	0.70	0.31

1 Values are least squares means of 13 samples per treatment ± the standard error.

2 FIdx: Fragmentation index, EAI: Emulsion activity index, ESI: Emulsion stability index.

3 Control: control diet (20.4% CP and 19.5% CP in the grower and finisher diets, respectively), CP-1.5%: −1.5% CP, CP-3.0%: −3.0% CP.

4 TRT is the *P*-value of the effect of treatment on analyzed traits; L and Q are the *P*-values of the linear and quadratic contrasts, respectively. C vs. CP is the *P*-value of the contrast comparing the average of the 2 experimental diets against the control diet. Means within a row lacking a common superscript differ at *P* < 0.05.

**Table 8:** Effect of dietary treatments on the proximate composition of breast meat.^1^

Trait	Treatments^2^			*P*-value^3^			
	Control	CP-1.5%	CP-3.0%	TRT	L	Q	C vs. CP
Dry matter, %	24.28 ± 0.18	24.15 ± 0.21	24.15 ± 0.21	0.87	0.69	0.84	0.66
Protein, %	20.91 ± 0.20	20.92 ± 0.22	21.10 ± 0.21	0.78	0.54	0.83	0.68
Lipid, %	1.88 ± 0.13	1.53 ± 0.15	1.56 ± 0.14	0.13	0.15	0.36	0.10

1 Values are least squares means of 13 samples per treatment ± the standard error.

2 Control: control diet (20.4% CP and 19.5% CP in the grower and finisher diets, respectively), CP-1.5%: −1.5% CP, CP-3.0%: −3.0% CP.

3 TRT is the *P*-value of the effect of treatment on analyzed traits; L and Q are the *P*-values of the linear and quadratic contrasts, respectively. C vs. CP is the *P*-value of the contrast comparing the average of the 2 experimental diets against the control diet. Means within a row lacking a common superscript differ at *P* < 0.05.

## DISCUSSION

Reducing dietary CP content in broiler's diets is one effective strategy to reduce nitrogen emissions (Lemme et al., 2019; Alfonso-Avila et al., 2022). A decrease of 3.0% in CP content during the grower and the finisher phases has no or limited consequences for broilers’ performance (de Rauglaudre et al., 2023), carcass yield, or BMY when AA profiles are adjusted to meet birds’ requirements (Belloir et al., 2017; Lambert et al., 2022). However, little is known about the effect of reduced CP content on breast meat quality, which is of interest for both the final consumers and the industry. Thus, this study investigated the implications of a moderate CP reduction in broiler diets supplemented with free limiting AAs to meet birds’ requirements on breast meat quality and yield.

### Effect of Reduced-CP Diets on Performance, Carcass Yield, and Meat Yield

Birds’ performance including weight gain, feed intake, and FCR over the whole experiment (0–35 d) were not influenced by the effect of the experimental diets despite a significant decrease in their N intake (−7.5 and −11.9% for the CP-1.5% and CP-3.0% group, respectively) compared with the control group over the same period. Belloir et al. (2017) conducted an experiment on male Ross broilers in which diets containing 19.0%, 17.5%, and 16.0% CP were fed to birds from 21 to 35 d. Similar to the present study, these authors did not find any significant differences between the 3 groups in terms of BW, weight gain, feed intake, or FCR. Belloir et al. (2017) formulated their experimental diets using dAA/dLys ratios defined by Mack et al. (1999) except for dArg/dLy and dThr/dLys that were adjusted to ratios defined by Rostango et al. (2011) to avoid Gly reduction when CP was reduced. They argued that when birds’ requirements in AA were met by supplementation with free AA, which was the case in our study (Table 2), CP content can be reduced by 2 to 3 percentage points without negative implications for birds’ performance. In a more recent report, Lambert et al. (2022) studied the effect of diets differing in lysine standardized ileal digestibility (SID Lys of 90% or 100% of the recommendations for the strain) and in SID Lys/CP ratio on the performance of male Ross 308 broilers during the grower and finisher phases. By increasing SID Lys/CP ratio to between 5.5% and 6.1%, these authors were able to reduce CP content to 17.1% in the grower diet and to 15.3% in the finisher diet. Lambert et al. (2022) showed that CP content could be reduced to 17% and to 15.3% in grower and finisher diets formulated at 90% of Aviagen recommendations for SID Lys without a negative impact on performance traits including BW, feed intake and FCR for the period from d 0 to d 35. In this same study, Lambert et al. (2022) found that when diets were formulated at 100% of Aviagen recommendations for SID Lys, the lowest achievable CP level without detrimental effects on traits of economic importance was 19% and 17% in the grower and finisher diet, respectively. These findings indicated that, at both levels of SID Lys, a moderate reduction in CP content (1.0–3.0%) was achievable without a degradation in birds’ performance similarly to findings from our study. Our findings and findings from the above-cited literature emphasized that only when the requirements in limiting AA are met, the CP content can be reduced by 1.0% to 3.0% without influencing growth or feed efficiency.

Reducing dietary CP content by up to 3.0% had no adverse impact on body weight at slaughter or on carcass yield, which are traits of economic importance. These findings are in line with other reports showing that reducing CP content to 17.8% during the grower phase and to 16.8% during the finisher phase did not alter final BW (van Harn et al., 2019) or carcass yield (van Harn et al., 2019; Brink et al., 2022) when essential AA requirements were maintained by supplementing diets with free AA. Similar results were also reported by Lambert et al. (2022) who showed that reducing dietary CP content to 17% during the grower phase and to 15.3% during the finisher phase was achievable without negative consequences on carcass yield when SID Lys was maintained at values very similar to those reported in our study (1.04% vs. 1.0% in the grower diet and 0.93% vs. 0.95% in the finisher diet).

Reducing dietary CP content coupled with a dietary supplementation with free AAs to meet birds’ requirements was associated with increased yield of the Pectoralis major muscle. Compared with the control group, a gain of 0.75 and of 1.08 percentage points in this trait was found in the CP-1.5% and CP-3.0% group, respectively. Consequently, a corresponding gain of 0.79 and of 1.2 percentage points was observed in BMY in the CP-1.5% and CP-3.0% group, respectively. As discussed above, feed intake was unchanged under the effect of reduced CP content; therefore, the increase in BMY could not be explained by differences in feed intake. All diets used in the current study were formulated to meet birds’ requirements of essential AA, including digestible lysine and threonine (Rostagno et al., 2017), the 2 most important AA for optimal breast and leg meat yield, respectively (Mehri et al., 2016). Under similar levels of essential AA in all treatment groups, the difference between the control and the reduced-CP diets in BMY could be partly explained by less indigestible protein reaching the hindgut when CP content is reduced, which can reduce the formation of harmful protein fermentation products such as biogenic amines (Qaisrani et al., 2015). Consequently, low CP diets could result in an enhanced digestibility of these essential AA leading to increased BMY. This statement is supported by a previous report showing that reducing dietary CP from 20.0% to 15.6% while maintaining SID Lys and SID AA/SID Lys at the ideal protein ratios in broilers’ diets was associated with an increased apparent digestibility coefficient of lysine and threonine in both the distal jejunum and the distal ileum at 35 d (Chrystal et al., 2020). Other carcass cuts were not influenced by the experimental diets, except for the wings yield that were slightly lower in the groups that received reduced-CP diets than in the control (−0.17 and −0.45 percentage points in the CP-1.5% and CP-3.0% group, respectively); these findings are similar to those from a previous report (van Harn et al., 2019). Taken together, our findings confirmed that CP content can be reduced by up to 3.0% in the grower and finisher phases without a negative impact on body weight at slaughter or on carcass and meat yield. However, these results can only be achieved when birds’ requirements in AA are adequately met.

### Effect of Reduced-CP Diets on Breast Meat Quality

Here, reducing dietary CP content in the range of 1.5 to 3.0% by partially replacing soybean meal with corn and canola meal while meeting birds’ requirements in limiting AA was associated with a tendency of the ultimate pH of the Pectoralis major muscle (PM-pHu) to increase. This aligns with a previous study that investigated reducing CP content from 19 to 16% while maintaining dLys at 0.9% during the finisher phase and reported an increase in the PM-pHu with decreased CP content (Belloir et al., 2017). These authors suggested that the reduced CP content could reduce excess AA that could potentially be used to synthesize and store energy in the Pectoralis major muscle. By reducing excess AA, reducing CP decreases glycogen stores in the muscle and subsequently increases the pHu. This explication is supported by findings from earlier studies investigating nutritional strategies to improve breast meat quality (Berri et al., 2008; Guardia et al., 2014). In their study, Berri et al. (2008) formulated diets with true digestible lysine (TD Lys) levels ranging from 0.83 to 1.13%. These diets were fed to male Ross 308 broilers during the finisher phase and resulted in a significant increase of the PM-pHu from 5.93 at 0.83% to 6.04 at 1.13% TD Lys. Berri et al. (2008) suggested that by increasing muscle growth and decreasing carcass fatness, higher levels of Lys could lead to lower glycogen storage in the Pectoralis major muscle and consequently to higher PM-pHu. The effect of excess AAs on PM-pHu was confirmed by the study of Guardia et al. (2014) who fed broilers with diets differing in their AA profiles and showed a significantly lower PM-pHu when broilers were fed, shortly before slaughter (33–36 d), a diet deficient in Lys (0.8%) with an excess amount of other AA (+6.0 to +12.0% in comparison to the control depending on the AA) compared with those fed a diet deficient in both Lys (0.8%) and other AA (-8.6 to -12.0% in comparison to the control depending on the AA). This relationship between PM-pHu and the levels of Lys and other AA was recently confirmed in the study of Belloir et al. (2019). These authors fed broilers with diets differing in AA profiles from 21 to 36 d and found the highest PM-pHu values (> 6.0) in breast meat from birds that received diets reduced in Lys (0.7%) and in Lys/AA ratios (-10.0%) relative to Lys/AA ratios defined by Mack et al. (1999). In addition, the lowest values of PM-pHu (< 5.8) in this study were found in breast meat of birds that received diets deficient in Lys (0.7%) with a 10.0% increase in Lys/AA ratios relative to Lys/AA ratios defined by Mack et al. (1999). These findings showed that PM-pHu was more responsive to changes in AA profile than in dietary CP content. This could explain the lack of a more pronounced difference in PM-pHu between groups in the current study as experimental diets were formulated with an AA profile similar to that of the control diet in both the grower and finisher phases.

The pHu of the Pectoralis major muscle is a major determinant of the final quality and processing ability of breast meat (Alnahhas et al., 2014). Extreme variations in muscle pH such as rapid or extended postmortem decline lead to extensive denaturation of muscle proteins, which then alters meat quality attributes including color and texture (Barbut et al., 2005; Bowker and Zhuang, 2015). Accordingly, the lack of differences in the lightness (L*) and the redness (a*) of breast fillets in the current study could be attributed to the lack of marked variations in the PM-pHu (Barbut et al., 2005; Le Bihan-Duval et al., 2008). This statement is supported by the lack of significant differences between treatments in myofibrillar and sarcoplasmic protein denaturation in the present study, as measured by their solubility. Despite the lack of significant changes in PM-pHu and in the functional properties of muscle proteins, yellowness (b*) of breast fillets decreased significantly with decreased CP content. The above-reported findings suggest that factors other than PM-pHu could have influenced b*.

The fragmentation index measures the fragmentation of myofibers and is an indirect measure of meat shear force, with higher values being associated with lower fragmentation and greater force required to shear muscle fibers (Thompson et al., 1987). The lack of between-treatment differences in this index is in coherence with the lack of pronounced differences in the shear force of breast fillets and supports the conclusion that reducing CP content in the range of 3.0% in the grower and finisher phases had no noticeable impact on meat tenderness when birds’ requirements in AA are adequately met through dietary supplementation with free AA. Water-holding capacity is also largely determined by variations in muscle pHu (Pietrzak et al., 1997; Huff-Lonergan and Lonergan, 2005), with increasing PM-pHu being associated with decreased cooking loss (Alnahhas et al., 2017). Although PM-pHu only tended to increase in reduced-CP diets, we saw a slight but significant decrease in cooking loss in CP-1.5% (−0.78 percentage points) and in the CP-3.0% diet (−1.75 percentage points) compared with the control group. The decrease in cooking loss with decreased CP content was independent from changes in the functionality of muscle protein; in the current study, the intra-treatment correlations between the total solubility of the myofibrillar and sarcoplasmic fractions and the cooking loss were not statistically significant (data not shown) which is in line with findings reported in the literature (Bowker and Zhuang, 2015). These authors analyzed breast fillets diverging in their pH at 6 and 24 h postmortem and showed that although overall solubility of sarcoplasmic and myofibrillar proteins was not correlated with changes in cooking loss, the denaturation of specific sarcoplasmic proteins such as glycogen phosphorylase was significantly correlated with cooking loss. They suggested that by precipitating on myofibrillar proteins, denatured glycogen phosphorylase increased cooking loss. Future studies should investigate changes in the relative abundance of glycogen phosphorylase and other sarcoplasmic proteins of interest for WHC in response to changes in dietary CP content to better understand the relationship between these parameters.

The effect of the dietary treatments on the emulsifying properties of the myofibrillar and sarcoplasmic fractions of breast muscle proteins was also investigated in our study. The EAI quantifies the ability of muscle proteins to interact with the water–oil interface and to form an emulsion system by preventing the coalescence of fat droplets, while the ESI measures the rate of the destabilization of the emulsion system over time through coalescence of these droplets (Chan et al., 2011; Bowker and Zhuang, 2016). The emulsifying properties of muscle proteins are highly influenced by muscle pH, which determines the protein's net charge and surface activity (Qiao et al., 2001; Padial-Domínguez et al., 2020). These properties are also influenced by protein solubility and concentration (Chan et al., 2011). Consequently, the lack of differences in the EAI and ESI reported in the present work for both fractions can be attributed to the lack of pronounced or significant differences between treatment groups in muscle pHu and in the solubility of the sarcoplasmic and myofibrillar fractions. These findings demonstrate that reducing dietary CP content in the range of 3.0% coupled with supplementing diets with unbound AA to meet birds’ requirements was not associated with significant changes in muscle protein functionality. Overall, our results showed that reducing dietary CP content by up to 3.0% during the grower and finisher phases of broiler chickens can be achieved without deteriorating the technological quality and processing ability of the breast meat as long as birds’ requirements in AA are adequately met.

Finally, the current analysis did not reveal any significant differences between the reduced-CP diets and the control diet in terms of muscle dry matter, protein content, or lipid content; values remained in the range of normal values as reported in the literature for broiler breast meat (Chen et al., 2016; de Oliveira et al., 2016). In this study, the abdominal fat percentage was not measured, but it is well established that when dietary CP content is reduced with a concomitant increase in corn incorporated in the diet, abdominal fat percentage increases as a result of the excess energy from dietary starch (Belloir et al., 2017; Chrystal et al., 2020; Lambert et al., 2022). However, in broiler chickens, intramuscular fat content is a genetically different trait that is not associated with abdominal fat (Zerehdaran et al., 2004). Therefore, even when abdominal fat increases in birds fed reduced-CP diets, it is not surprising that the intramuscular fat content of breast meat remains unchanged.

## CONCLUSIONS

The current study was conducted to provide new and original data on the effect of reducing dietary CP content in broilers’ diets on the technological quality of breast meat and on the functional properties of breast muscle proteins. According to the findings, reducing CP content in the range of 3.0% can be achieved without noticeable consequences for breast meat quality traits when birds’ AA requirements are adequately met through dietary supplementation with free AA. Furthermore, this study confirmed previous results that reducing CP in the range of 3.0% was not associated with undesirable impact on traits of economic importance such as body weight and feed efficiency when the requirements in AA are sufficiently met. One of the most interesting findings from this work was the increased BMY associated with decreasing CP content; this was likely caused by improved digestibility of AAs in the intestinal tract of birds receiving these diets. Moreover, CP content can be reduced without altering the nutritional value or the image of broiler breast meat as a healthy alternative to red meat, because protein and fat content in breast meat remained in their normal range. In light of these findings, it can be concluded that reducing CP content by 3.0% in the grower and finisher diets of broiler chickens to reduce emissions and improve the environmental sustainability of broiler production could be deployed in practice without notable impact on the economic sustainability of this production, as long as the birds’ AA requirements are adequately met.
